# Progranulin concentration in relation to bone mineral density among obese individuals

**DOI:** 10.20945/2359-3997000000022

**Published:** 2018-03-23

**Authors:** Alireza Milajerdi, Zhila Maghbooli, Farzad Mohammadi, Banafsheh Hosseini, Khadijeh Mirzaei

**Affiliations:** 1 Tehran University of Medical Sciences Tehran University of Medical Sciences Endocrinology and Metabolism Clinical Sciences Institute Tehran Iran Endocrinology and Metabolism Clinical Sciences Institute, Tehran University of Medical Sciences, Tehran, Iran; 2 Tehran University of Medical Sciences Tehran University of Medical Sciences School of Nutritional Sciences and Dietetics Department of Community Nutrition Tehran Iran Department of Community Nutrition, School of Nutritional Sciences and Dietetics, Tehran University of Medical Sciences (TUMS), Tehran, Iran

**Keywords:** Bone mineral density, progranulin, obesity, osteopenia, cytokine

## Abstract

**Objective:**

Adipose tissue, particularly visceral adipose tissue, secretes a variety of cytokines, among which progranulin is a glycoprotein related to the immune system. Along with other secreted proteins, progranulin may be associated with bone mineral density. The aim of this study was to find out whether there are associations between the progranulin and bone mineral density among obese people.

**Subjects and methods:**

This cross-sectional study was conducted on 244 obese participants (aged 22-52). Serum progranulin, high sensitive C-reactive protein, oxidised-low dencity lipoprotein, tumor necrosis factor-α, parathormone, vitamin D, and interleukins of 1 β, 4, 6, 10, 13, and 17 concentrations were measured. Anthropometric measurements, body composition and bone mineral density were also assessed.

**Results:**

Serum progranulin was directly associated with interleukin-6 and interleukin-1β, while it had a negative association with interleukin-17 and tumor necrosis factor-α. We also observed a statistically significant direct association between progranulin concentration and visceral fat, abdominal fat, waist, abdominal and hip circumferences, hip T-score, and Z-score and T-score for the lumbar region. A partial correlation test has also shown a significant positive correlation regarding serum progranulin and the hip Z-score. Moreover, progranulin level is inversely associated with ospteopenia (P = 0.04 and CI: 0.17,0.96).

**Conclusion:**

Our study revealed that central obesity may be related to increased progranulin concentration. In addition, progranulin concentration was directly related to bone formation parameters, which indicates the protective effects of progranulin on bone density. Further studies are needed to clarify the exact mechanisms underlying these associations.

## INTRODUCTION

Compelling evidence indicates that obesity is associated with inflammation and immune system function (1,2). Due to the function of adipose tissue as an endocrine gland which secretes several cytokines, this association has frequently been attributed to central obesity (3). Indeed, some studies have regarded inflammation as a risk factor for osteoporosis (4,5). However, the precise mechanism underlying the role of inflammation as well as the association between obesity, inflammation and osteoporosis has not yet been clarified. Some data suggest that cytokines secreted from adipose tissue may play a major role in osteoporosis (6). Osteoporosis is considered as one of the most serious chronic diseases in the present century (7). According to the international definition, osteoporosis defined as a 2.5 Standard Deviation (SD) reduction in Bone Mineral Density (BMD) (8,9).

Progranulin (PGRN) is a cytokine that is secreted from adipose tissue, and also, is a secretory 593-amino acid glycoprotein with a widespread expression in different cells, such as immune system cells (10). PGRN is also regarded as a growth factor, similar to IGF-1, with inflammatory properties (11). There are some studies that suggested PGRN concentration is associated with the extent of visceral adiposity (12,13). It seems that PGRN can activate some inflammatory pathways (14) and, as mentioned, this can affect BMD (15) and facilitate the development of osteoporosis. Furthermore, a recent study by Romanello and cols. has suggested a proliferative and pro-survival effect of PGRN on osteocyte-like cells. The research demonstrated that PGRN can induce phosphorylation of mitogenactivated protein kinase in both HOBIT and osteocytic cells. Moreover, the authors reported that Risedronate, a bisphosphonate drug which has been widely used in the treatment of osteoporosis, induces the expression as well as the secretion of PRGN in the HOBIT secretome. These findings suggested the possible role of PGRN in osteoblast/osteocyte biology (16).

The aim of this study was to find out whether there are associations between progranulin and bone mineral density among obese people.

## MATERIALS AND METHODS

### Study population

In this cross sectional study, 244 class I and II obese (30 ≤ BMI < 40 kg/m^2^) participants (22 to 52 years old) were recruited from Shariati hospital. The study protocol was approved by the ethics committee of the Endocrinology and Metabolism Research Center of Tehran University of Medical Sciences (TUMS) with the following identification: 90-03-27-14619. The inclusion criteria namely were having a BMI in the range of 30-39.99kg/m^2^, and being aged from 22-52. Exclusion criteria were defined as having any history of inflammatory conditions or inflammatory diseases, cardiovascular disease, diabetes mellitus, thyroid diseases, cancer or malignancies, hypertension or hypotensive drug abuse, hepatic, heart, or renal disease, chronic or acute infections, smoking, drug or alcohol abuse, and pregnancy. Each participant was completely informed regarding the study protocol and provided a written and informed consent form before taking part in the study.

### Laboratory measurements

All blood samples were collected from 8:00 to 10:00 a.m. after an 8-12 hours fast at the EMRC laboratory in Shariati hospital of TUMS. To collect serums, blood samples were centrifuged for 10 minutes at 3000 rpm. Serum samples were aliquoted and stored at -80°C until they were analyzed. Serum high sensitive C-reactive protein (hsCRP), as a sensitive marker of inflammation, was measured by an imonoturbidimetric assay (Randox laboratories kit, Hitachi 902). Serum concentrations of adipokines (including interleukins of 1 β, 4, 6, 10, 13, and 17) were measured in triplicate and 10 replicates per EIA plate under internal quality controls. Serum concentration of interleukin 6 (IL-6) was analyzed by EIA kit (Enzo Life Sciences, Inc. Sensitivity: 3.75 pg/mL; inter-assay variability: 3.7%; intra-assay variability: 3.9%). Serum concentration of interlukin 4 (IL-4) was also assessed by EIA kit (Enzo Life Sciences, Inc. Sensitivity: < 2 pg/mL; in intra CV was 4.3% and interCV was 4.7%). TNF-α concentration was determined by EIA kit (Enzo Life Sciences, Inc. Sensitivity: 8.43 pg/mL; inter-assay variability: 6%; intra-assay variability: 3.6%). Serum PGRN concentration was measured by ELISA kit (AdipoGen; Seoul, South Korea. Sensitivity: 32 pg/mL; inter-assay variability: 4.7%; intra-assay variability: 3.79%) under internal quality controls (17).

### Anthropometric measurement

Weights and heights were measured with participants wearing light clothes and without shoes. Weight was measured using a digital scale (Sega 707, Hamburg, Germany) to the nearest 0.1 kg. Height was measured using a stadiometer (Seca, Hamburg, Germany) to the nearest 0.1 cm. Body mass index (BMI) was calculated using the “weight(kg)/height^2^(m^2^)” equation. Waist circumference (WC) was measured in the middle point of the iliac crest and ribcage.

### Body composition analysis

Participant body composition was assessed by *Body Composition Analyzer BC-418MA – Tanita* (*United Kingdom*). This Bioelectrical Impedance Analyzer (BIA) sends out a very weak electric current across the body to measure its electrical resistance. Before assessing body composition, the manufacturer's instructions were followed to ensure accurate assessment. Participants were asked not to exercise vigorously, put aside any electrical device (mobile phone, etc.), or to intake excessive fluid or food. As changes in bodywater distribution and body temperature can have a major impact on measurements, they were performed in the morning in a fasting condition (always urinating before taking measurements, etc.) to get a more accurate measurement every single time. To prevent inaccurately low body fat percentage measurements and other measurement errors, both arms were always held straight down when taking measurements. The device calculates the body fat percentage, fat mass and fat-free mass, and predicts the muscle mass on the basis of data obtained by dual-energy X-ray absorptiometry using bioelectrical impedance analysis (18).

### BMD measurement

In this study, BMD was measured by the Dual Energy X-ray Absorptiometery (DEXA) method at the hip and lumbar spine (vertebra L2-L4). The average coefficient of variation (CV) for measuring BMD in our device was 1.04%. According to the World Health Organization (WHO) standard, normal bone mass was defined as BMD ≥ -1 standard deviation (SD), osteopenia as -1 < BMD <-2.5 SD, and osteoporosis as BMD ≤ -2.5 SD. Osteoporosis was diagnosised based on the T-score (19).

### Statistical analysis

The study population was divided into two groups based on median PGRN concentration (< 113.30 and ≥ 113.30 pg/mL), then the study variables were compared among the two groups using an independent T-test. The association between serum PGRN concentration and BMD measurements was examined through a partial correlation test after adjusting for weight and fat mass. The level of statistical significance was set to < 0.05 All statistical analysis was performed using SPSS version 16.0 (Chicago, IL).

## RESULTS

The particpants’ mean (±SD) of age, height, BMI, and weight were 39.12 ± 11.90 years, 162.42 ± 8.80 cm, 35.32 ± 3.98 kg/m^2^, and 93.68 ± 14.75 kg, respectively. The mentioned variables were 37.10 ± 12.77 years, 176.44 ± 6.89 cm, 35.30 ± 3.49 kg/m^2^, and 108.42 ± 15.74 kg respectively, in men, and 39.60 ± 11.68 years, 159.87 ± 6.38 cm, 35.33 ± 4.10 kg/m^2^, and 90.15 ± 12.14 kg, respectively in women. From 244 participants, 68 subjects (27.86%) were osteopenic and 176 individuals (72.14%) had normal BMD. The population characteristics, body composition, BMD, and laboratory measurements of participants are summarized in Table 1. As shown in the table, mean serum parathyroid hormone (PTH) and vitamin D (VitD) concentrations of participants were above and within the normal ranges respectively (10-55 pg/mLfor PTH and 30-74 ng/mL for VitD). The visceral fat rating showed that central obesity may be more serious in men compared to women (Table 1). Additionally, the DEXA assay showed an osteopenic condition only in the lumbar spine (L2-L4) of women, when BMD was expressed as mean ± (SD) T-score (T-score < -1).

**Table 1:** Population characteristics, body composition, bone mineral density and laboratory measurements of the participants

Variables	Men (n = 57)	Women (n = 187)
**Anthropometry:**		
Age (years)	37.10 ± 12.77	39.60 ± 11.68
Weight (kg)	108.42 ± 15.74	90.15 ± 12.14
Height (21)	176.44 ± 6.89	159.87 ± 6.38
BMI (kg/cm^2^)	35.30 ± 3.49	35.33 ± 4.10
Fat percent (%)	30.11 ± 4.23	42.43 ± 4.75
Fat mass (kg)	32.98 ± 8.22	38.48 ± 8.21
Fat-free mass (kg)	75.41 ± 9.53	51.66 ± 6.57
Visceral fat rating (kg)	14.24 ± 4.05	9.99 ± 2.46
Trunk fat percent (%)	20.80 ± 4.67	18.75 ± 4.37
**Biochemistry characteristics:**		
Progranulin (pg/mL)	119.22 ± 30.13	120.86 ± 44.02
Vitamin D (ng/mL)	32.43 ± 4.50	38.28 ± 36.39
Hs-CRP (mg/L)	2.35 ± 2.32	4.75 ± 5.86
Ox-LDL (U/dL)	556.31 ± 58.85	583.02 ± 85.54
TNF-α (pg/mL)	13.68 ± 30.92	7.67 ± 14.01
PTH (pg/mL)	84.74 ± 54.19	89.89 ± 49.90
IL-1β (pg/mL)	0.01 ± 0.00	0.01 ± 0.00
IL-4 (pg/mL)	1.47 ± 0.86	1.81 ± 1.07
IL-6 (pg/mL)	30.28 ± 18.99	24.09 ± 20.66
IL-10 (pg/mL)	31.85 ± 38.65	14.12 ± 15.44
IL-13 (pg/mL)	32.35 ± 33.60	41.61 ± 30.41
IL-17 (pg/mL)	0.28 ± 0.13	0.95 ± 1.33
**Bone densitometry:**		
Hip BMD	1.17 ± 0.16	1.08 ± 0.16
Hip T-score	0.64 ± 1.27	0.58 ± 1.10
Hip Z-score	0.18 ± 1.03	0.22 ± 0.99
Lumbar BMD	1.24 ± 0.18	1.19 ± 0.16
Lumbar T-score	0.24 ± 1.58	-0.10 ± 1.17
Lumbar Z-score	-0.48 ± 1.45	-0.68 ± 1.13

BMI: body mass index; Hs-CRP: high sensitive C-reactive protein; TNF-α: tumor necrosis factor-α; IL: interleukin; Ox-LDL: oxidized-low density lipoprotein; PTH: parathormone; BMD: bone mineral density.

### Association between PGRN, anthropometric measures, and body composition

Our analysis revealed that mean BMI, fat percentage, fat mass, fat free mass, visceral fat, trunk fat, waist circumference, abdominal and hip circumferences were greater in the high serum PGRN concentration group compared to the low serum PGRN concentration one (Table 2). However, the association was statistically significant for visceral fat, trunk fat, waist, abdominal and hip circumferences (p < 0.05). We also found a higher mean of age (p = 0.17) and total body water (p = 0.28) in the high serum PGRN concentration group, which were not significant. In addition, a difference in the Central Adiposity Index (CAI) lower than 25 and above 75 centile value was seen in participants, with the following results reported: waist circumference was 95.83 cm ± 3.64 SE and 98.16 ± 1.97 in participants in the lower than 25 and above 75 centile for progranulin value, respectively, which was not significant (P-value = 0.12). Results also highlighted values of WHR (0.86 ± 0.01 and 0.87 ± 0.01 and P-value = 0.75), visceral fat (6.30 ± 0.66 and 7.88 ± 0.41 and P-value < 0.0001) and BMI (27.85 ± 1.12 and 31.06 ± 0.65 and P-value = 0.001) in lower than 25 and above 75 centile for progranulin value, respectively.

**Table 2:** Anthropometric measures and body composition between groups with low and high concentrations of PGRN

Variables	Relative PGRN concentration (n = 244)		P value
	Low concentration (n = 122)	High concentration (n = 122)	
Age (years)	38.08 ± 12.86	40.16 ± 11.22	0.17
BMI (kg/cm^2^)	34.54 ± 3.85	35.12 ± 4.44	0.27
Fat percent (%)	40.79 ± 6.56	40.86 ± 6.32	0.93
Fat mass (kg)	36.32 ± 8.86	37.40 ± 9.00	0.34
Fat-free mass (kg)	52.50 ± 9.53	53.76 ± 8.98	0.28
Visceral fat rating (kg)	9.56 ± 2.94	10.45 ± 2.71	**0.01**
Trunk fat percent (%)	17.57 ± 5.42	19.13 ± 4.39	**0.01**
Waist circumference (21)	97.00 ± 9.99	101.56 ± 9.46	**< 0. 001**
Abdominal circumference (21)	111.42 ± 8.88	116.45 ± 10.56	**< 0. 001**
Hip circumference (21)	114.00 ± 7.09	118.38 ± 11.55	**< 0.001**
TBW (%)	38.42 ± 6.99	39.36 ± 6.57	0.28

BMI: body mass index; TBW: total body water.

### Association between PGRN and other cytokines

According to an independent T-test, mean serum concentrations of IL-1β, IL-13, Il-10, Il-6, and Il-4 were greater in those in the high serum PGRN concentration group (Table 3), while mean serum levels of IL-17, TNF-α, and hs-CRP were lower in the high PGRN concentration group compared to the low serum PGRN concentration one. However, it should be noted that these associations were statistically significant only for IL-1β, IL-17, IL-6, and TNF-α (p < 0.05).

**Table 3:** Cytokine concentrations between groups with low and high concentrations of PGRN

Variables	Relative PGRN concentration (n = 244)		P value
	Low concentration (n = 122)	High concentration (n = 122)	
Hs-CRP (mg/L)	4.91 ± 4.98	4.73 ± 6.99	0.81
TNF-α (pg/mL)	10.67 ± 19.68	3.53 ± 2.09	**< 0.001**
IL-1β (pg/mL)	0.01 ± 0.00	0.02 ± 0.01	**< 0.001**
IL-4 (pg/mL)	2.02 ± 1.35	2.21 ± 0.79	0.18
IL-6 (pg/mL)	16.68 ± 11.80	28.29 ± 28.15	**< 0.001**
IL-10 (pg/mL)	12.43 ± 12.21	15.51 ± 22.45	0.18
IL-13 (pg/mL)	41.83 ± 29.24	47.01 ± 31.44	0.18
IL-17 (pg/mL)	1.31 ± 1.55	0.45 ± 0.61	**< 0.001**

Hs-CRP: high sensitive C-reactive protein; TNF-α: tumor necrosis factor-α; IL: interleukin.

### Association between PGRN and bone health variables

**Figure 1:**
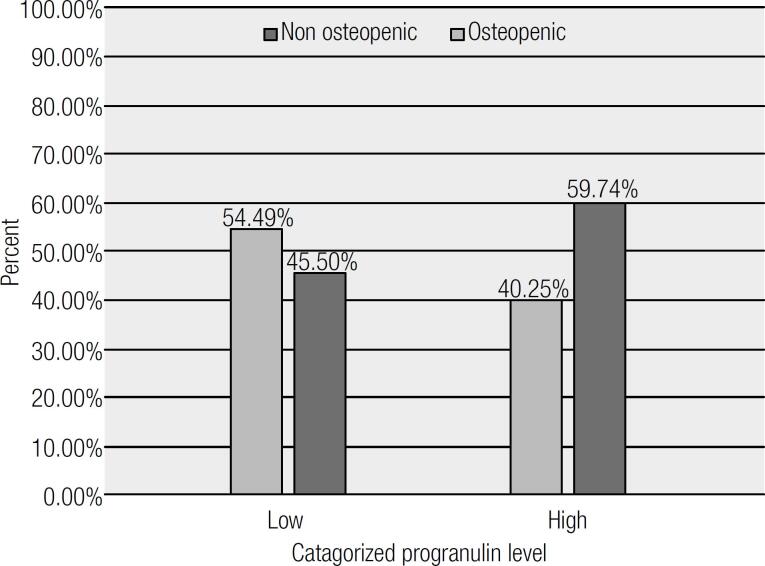
Variety of osteopenia by categorized progranulin level.

According to an independent T-test, mean hip BMD and hip T-score and Z-score, as well as T-score and Z-score for the lumbar spine (L2-L4 vertebra) were greater in the high serum PGRN concentration group (Table 4). However, it should be noted that these associations were statistically significant only for hip T-score and Z-score and the lumbar T-score (p < 0.05) and was marginally significant considering total BMD (p = 0.05). Moreover, a partial correlation between serum PGRN concentration and BMD measurements adjusted for fat mass, indicated a significant positive correlation with hip Z-score (r = 0.35, p < 0.05). However, after factoring in weight, none of the observed correlations were significant (Table 5, part A). Additionally, a binary regression analysis was done to strengthen our findings, (as shown in Table 5, part B). After adjustment for age and BMI, the PGRN level was strongly and inversely associated with osteopenia (P = 0.04 and CI: 0.17,0.96). Figure 1 demonstrates that there was a significantly lower number of osteopenic patients in the high serum PGRN concentration group.

**Table 4:** Bone mineral density measurements between groups with low and high concentrations of PGRN

Variables	Relative PGRN concentration (n = 244)		P value
	Low concentration (n = 122)	High concentration (n = 122)	
Vitamin D (ng/mL)	32.76 ± 35.92	28.69 ± 13.99	0.24
PTH (pg/mL)	92.00 ± 56.76	105.74 ± 63.04	0.09
Hip BMD	1.07 ± 0.23	1.12 ± 0.17	**0.05**
Hip T-score	0.18 ± 0.79	1.05 ± 1.50	**< 0.001**
Hip Z-score	-0.13 ± 0.89	0.68 ± 1.30	**< 0.001**
Lumbar BMD	1.15 ± 0.13	1.18 ± 0.16	0.10
Lumbar T-score	-0.41 ± 1.16	-0.08 ± 1.36	**0.04**
Lumbar Z-score	-0.85 ± 1.07	-0.61 ± 1.38	0.13

BMD: bone mineral density; PTH: parathormone.

**Table 5:** A: Partial correlation between PGRN concentration and bone mineral density measurements

	Adjusted for	Hip BMD	Hip T-score	Hip Z-score	Lumbar BMD	Lumbar T-score	Lumbar Z-score
**Progranulin concentration (pg/mL)**	r						
	Weight	0.21	0.32	0.33	0.24	0.24	0.26
	P value	0.25	0.75	0.65	0.19	0.18	0.15
	r						
	Fat mass	0.19	0.33	0.35	0.22	0.23	0.26
	P value	0.30	0.06	**0.04**	0.22	0.21	0.14

BMD: bone mineral density.

**B:** Binary regression model for analyzing the relationship between Progranulin Concentration and risk of osteopenia in obese people

PGRN	Crude model			Model 1			Model 2			Model 3		
	B ± SE	CI	P	B ± SE	CI	P	B ± SE	CI	P	B ± SE	CI	P
	-0.47 ± 0.32	0.33, 1.69	0.14	-0.52 ± 0.33	0.30, 1.30	0.11	-0.90 ± 0.99	0.17, 0.96	**0.04**	-0.81 ± 0.44	0.81, 1.07	0.07

Model 1: Adjusted for age; Model 2: Adjusted for age and BMI; Model 3: Adjusted for age, BMI and gender; PGRN: Progranulin

Additionally, we found that serum PTH was greater in the high serum PGRN concentration group (p = 0.09), while vitamin D was lower in this group, in comparision with those who had lower serum concentrations of PGRN (p = 0.24). However, none of them were statistically significant.

Meanwhile, comparing the prevalence of osteopenia among these two groups indicated that the osteopenic patients had considerably lower serum PRGN concentrations (Figure 1).

## DISCUSSION

In the current study, a significant association was found between PGRN concentration and the serum levels of some cytokines, which can be explained by the regulatory roles of PGRN on signaling pathways. To illustrate this, it can be observed that serum PGRN was directly associated with the levels of serum IL-1β and Il-6, while it was inversely related to IL-17 and TNF-α serum levels. Results also showed significant associations between PGRN concentration and visceral and trunk fat. Increased hip, waist and abdominal circumferences were also observed in the higher concentration PGRN group. Furthermore, high PGRN was related to higher hip T- and Z-score and also lumbar T-score.

These findings agreed with Zhang and cols.'s study, which found a significant linear correlation between PGRN concentration and IL-6 serum levels in patients with primary Sjögren's syndrome (20). Frampton and cols. also have reported that Il-6 can activate the ERK1/2/RSK1/C/EBPβ pathway and PGRN synthesis as a consequence (21). Furthermore, several studies have shown that PGRN may antagonize TNF-α by the activation of its receptors, therefore PGRN may have some anti-inflammatory properties (22). Studies have also regarded TNF-α as an inhibitor of osteoblast differentiation, as well as an activator of osteoclastogenesis (23).

According to previous studies, IL-6 and IL-1β induce bone resorption and inhibit bone formation (24). Although it has been widely reported that IL-17 mediates diverse inflammatory processes, its effects on bone resorption has recently been documented (25). It seems that IL-17 and TNF-α synergically stimulate bone resorption (26).

This study demonstrated a significant linear association between PGRN concentration and central obesity parameters including visceral fat, abdominal fat, waist, abdominal and hip circumferences. Along the same lines as these findings, previous studies have found that the PGRN gene expresses in macrophages existing in fat tissues, especially visceral fat (12,13). Youn and cols. reported that PGRN concentration was significantly associated with central and general obesity parameters, which can be described by stimulating omental adipose tissue macrophage infiltration by PGRN (13). Pradeep and cols. measured PGRN concentrations in serum and gingival crevicular fluid of 40 patients suffering from chronic periodentitis with and without obesity. The authors reported that the serum PGRN concentration was higher in both serum and gingival crevicular fluid in obese periodentitic patients; which can indicate that inflammation related to periodentitis and obesity may also be associated with PGRN concentration (27). In agreement with these findings, Hossein-Nezhad and cols. demonstrated an association between BMI and central obesity with PGRN gene expression and circulation levels, which revealed that PGRN is related to obesity through *glucose homeostasis* and metabolism regulation (28).

Since we observed a linear association between PGRN concentration and central obesity parameters, changes in the secretion of cytokines may be attributed to adipose tissue expansion. Similarly, Zizza and cols.'s study found a negative association between IL-17 and visceral obesity (29). Furthermore, Mohamed-Ali's study showed that subcutaneous fat was associated with IL-6 but not with TNF-α (30).

Results from previous studies have indicated that visceral fat, as well as subcutaneous fat, as measured by computer tomography scan, and BMI have a negative association with bone density. More importantly, correlations regarding visceral fat and decreased bone density remained statistically significant even after adjustment for age, sex, and BMI (31). This study's findings demonstrate PGRN's effect on osteogenesis, as can be seen in the hip T-score and Z-score and T-score for the lumbar vertebra, which were significantly associated with PGRN concentration. Similarly to this study, Romanello and cols. demonstrated the proliferative and pro-survival effects of PRGN on osteocyte-like cells (16). Likewise, a recent study by Oh and cols. indicated a new regulatory axis by which PGRN may induce osteoclastogenesis. This axis is regarded as the (RANKL)/RANK axis, and PGRN may induce this pathway by stimulating PIRO expression (32). Documented results showed that recombinant human PGRN can induce phosphorylation of mitogenactivated protein kinase in both HOBIT and osteocytic cells and induce cell proliferation and survival. Moreover, they found that Risedronate, a widely used bisphosphonate drug in the treatment of osteoporosis, can induce the expression and secretion of PGRN in the HOBIT secretome (16). These findings have shown the probable preventive effects of PGRN on osteoporosis by modulating bone loss. In addition, results from previous studies reported that PGRN growth factor enhances chondrocyte differentiation and endochondral ossification by regulating BMP-2 and TNF signaling, therefore PGRN injections may play an important role in bone healing, particularly in fracture conditions (33). A disadvantage of the present cross-sectional study is that it does not allow definite conclusions to be made regarding cause and effect. PGRN might affect BMD or vice versa.

In conclusion, the findings of this study showed that central obesity expansion is associated with increased PGRN concentration. PGRN has some paradoxical relation with the levels of cytokine secretion, in that it has a direct association with the secretion of IL-1β and IL-6, while it inhibits the secretion of IL-17 and TNF-α. According to the aforementioned mechanisms, these changes in cytokine secretion have both degenerative and protective effects on bone structure and BMD. Aditionally, the association between obesity and bone mineral density has been demonstrated by several studies, though its effects depend on the definition of obesity; if obesity is regarded as increased body fat levels, it can be considered as a risk factor for a lower BMD, which can be affected by increased adipokines, subsequently causing a lower BMD (34). However, obesity seems to play its role as a protective factor against osteoporosis if it is defined as an increase in body weight, which can be explained by a higher level of circulating estradiol, increased peak bone mass and greater gravitational load (35). The direct observed association between PGRN concentration and bone formation parameters indicates that PGRN may have some bone-protective effects through various mechanisms other than cytokine secretion regulation. Further studies are needed to address the cellular and molecular mechanisms of both general and central obesity, as well as PGRN's effects on bone formation and absorption.
